# Gene expression profile suggests that pigs (*Sus scrofa*) are susceptible to *Anaplasma phagocytophilum* but control infection

**DOI:** 10.1186/1756-3305-5-181

**Published:** 2012-08-30

**Authors:** Ruth C Galindo, Nieves Ayllón, Katja Strašek Smrdel, Mariana Boadella, Beatriz Beltrán-Beck, María Mazariegos, Nerea García, José M Pérez de la Lastra, Tatjana Avsic-Zupanc, Katherine M Kocan, Christian Gortazar, José de la Fuente

**Affiliations:** 1 Instituto de Investigación en Recursos Cinegéticos IREC-CSIC-UCLM-JCCM, Ronda de Toledo s/n, Ciudad Real, 13005, Spain; 2 Institute of Microbiology and Immunology, Medical Faculty, Zaloska 4, Ljubljana, 1000, Slovenia; 3 Centro de Vigilancia Sanitaria Veterinaria (VISAVET), Facultad de Veterinaria, Universidad Complutense de Madrid, Madrid, 28040, Spain; 4 Department of Veterinary Pathobiology, Center for Veterinary Health Sciences, Oklahoma State University, Stillwater, OK, 74078, USA

**Keywords:** Anaplasmosis, Genetics, Pig, Wild boar, Genomics, Immune response

## Abstract

**Background:**

*Anaplasma phagocytophilum* infects a wide variety of hosts and causes granulocytic anaplasmosis in humans, horses and dogs and tick-borne fever in ruminants. Infection with *A. phagocytophilum* results in the modification of host gene expression and immune response. The objective of this research was to characterize gene expression in pigs (*Sus scrofa*) naturally and experimentally infected with *A. phagocytophilum* trying to identify mechanisms that help to explain low infection prevalence in this species.

**Results:**

For gene expression analysis in naturally infected pigs, microarray hybridization was used. The expression of differentially expressed immune response genes was analyzed by real-time RT-PCR in naturally and experimentally infected pigs. Results suggested that *A. phagocytophilum* infection affected cytoskeleton rearrangement and increased both innate and adaptive immune responses by up regulation of interleukin 1 receptor accessory protein-like 1 (*IL1RAPL1*), T-cell receptor alpha chain (*TCR-alpha*), thrombospondin 4 (*TSP-4*) and Gap junction protein alpha 1 (*GJA1*) genes. Higher serum levels of IL-1 beta, IL-8 and TNF-alpha in infected pigs when compared to controls supported data obtained at the mRNA level.

**Conclusions:**

These results suggested that pigs are susceptible to *A. phagocytophilum* but control infection, particularly through activation of innate immune responses, phagocytosis and autophagy. This fact may account for the low infection prevalence detected in pigs in some regions and thus their low or no impact as a reservoir host for this pathogen. These results advanced our understanding of the molecular mechanisms at the host-pathogen interface and suggested a role for newly reported genes in the protection of pigs against *A. phagocytophilum*.

## Background

*Anaplasma phagocytophilum* (Rickettsiales: Anaplasmataceae) is a tick-borne pathogen that infects a wide range of hosts including humans and wild and domestic animals [1,2]. *A. phagocytophilum* is the causative agent of human, equine and canine granulocytic anaplasmosis and tick-borne fever in ruminants [1,3,4]. In Europe, *A. phagocytophilum* is the most widespread tick-borne infection in animals with an increasing incidence in humans [5-10]. *A. phagocytophilum* is transmitted by *Ixodes* spp., but other tick species may subsequently also prove to be vectors [11,12]. Evidence suggests that persistent infections occur in domestic and wild ruminants, which can then serve as reservoir hosts [1,9]. The broad geographic distribution and the clinical and host tropism diversity of *A. phagocytophilum* strains suggest the presence of complex infection-transmission networks that may influence the epizootiology of the disease [13].

*A. phagocytophilum* has been reported with low prevalence in wild pigs (*Sus scrofa*) in the Czech Republic [14] and Slovenia [15]. Recently, 12% prevalence of was detected in wild boar in Poland [16]. In Slovenia and Poland, the *A. phagocytophilum* gene sequences found in wild pigs were identical to that found in humans and *I. ricinus* ticks [15,16]. In Sicily, evidence suggested that *A. phagocytophilum* infection might occur in pigs [17]. In south-central Spain, where *I. ricinus* are scarce [18], *Anaplasma* spp. has not been reported in wild boar [13,19,20], although other tick species feeding on wild boar were positive for *A. phagocytophilum* DNA [12]. Recently, 16S rDNA but not *p44*/*msp2* genotypes identical to *A. phagocytophilum* were found with low prevalence in wild boar in Japan [21] but a survey in Mississippi, United States, failed to detect pathogen DNA in feral pigs [22]. These results suggested that wild pigs might play a role in the epizootiology of *A. phagocytophilum* by serving as a natural reservoir host in some regions only.

Infection with *A. phagocytophilum* has been shown to modify the host cell gene expression. The gene expression profile has been characterized in human cells [23-28] and sheep [29] infected with *A. phagocytophilum*. As shown by recent studies in sheep [29], gene expression profile in response to *A. phagocytophilum* infection may differ between human cells and ruminant hosts. These differences may be the result of species-specific differences and/or the effect of different pathogen strains [2,29].

The objective of this study was to characterize gene expression profiles emphasizing on immune response genes in wild and domestic pigs in response to *A. phagocytophilum* using a combination of microarray hybridization and real-time RT-PCR. These results will expand current information on the mammalian host response to *A. phagocytophilum* infection and contribute to the overall understanding of the molecular mechanisms involved in pathogen infection, multiplication and persistence.

## Materials and methods

### Experimental design and rationale

The finding of wild pigs naturally infected with *A. phagocytophilum* in Slovenia suggested that this pathogen might also infect pigs, thus probably affecting gene expression in this species. The genes differentially expressed in response to *A. phagocytophilum* infection were first characterized in wild pigs naturally infected with *A. phagocytophilum* by microarray hybridization and real-time RT-PCR. The differentially expressed immune response genes were then further characterized in domestic pigs experimentally infected with *A. phagocytophilum* under controlled experimental conditions.

### Wild pigs and sample preparation

Buffy coats were prepared from blood samples collected from adult (≥1 year-old) wild pig males hunter-killed during 2007 in Kočevje–Šubičeva and Kostel–Delač, Slovenia. Total DNA and RNA were extracted using MagneSil KF genomic DNA (Promega, Madison, WI, USA) and TRIzol Reagent (Invitrogen, Life Technologies Corporation, Carlsbad, CA, USA), respectively according to manufacturer’s instructions. The DNA was used to test for *A. phagocytophilum* infection using 16S rDNA and *groESL* PCRs and sequence analysis as previously reported [15]. Three of the 113 pigs analyzed tested positive for the presence of *A. phagocytophilum* DNA and were selected for further analysis. Control Buffy coats were prepared from uninfected adult wild pig males hunter-killed in south-central Spain where pigs are not infected with *Anaplasma* spp. [13,19,20]. Control animals tested negative in the *A. phagocytophilum* 16S rDNA and *groESL* PCRs. All animals tested negative for other pathogens commonly found in wild pigs such as *Mycobacterium bovisBrucella suis*, Aujeszky´s Disease Virus (ADV) and porcine circovirus type 2 (PCV2).

### Microarray hybridization and analysis

Total RNA from wild pigs was characterized using the Experion™ Automated Electrophoresis System (Bio-Rad, Hercules, CA, USA) in order to evaluate the quality and integrity of RNA preparations. One RNA sample from infected animals did not have the quality required for microarray hybridization. Therefore, two samples from infected animals were selected for microarray hybridization analysis together with three RNA samples from uninfected control animals. To obtain a comprehensive gene expression profile in response to *A. phagocytophilum* infection, the GeneChip® Porcine Genome Array was used, which contains 23,937 probe sets that interrogate approximately 23,256 transcripts from 20,201 *S. scrofa* genes (Affymetrix, Santa Clara, CA, USA; http://www.affymetrix.com/products_services/arrays/specific/porcine.affx). Two μg total RNA were labeled using the GeneChip® HT IVT Labeling Kit (Affymetrix). The images were processed with Microarray Analysis Suite 5.0 (Affymetrix). Raw expression values obtained directly from CEL files were preprocessed using the RMA method [30], a three-step process which integrates background correction, normalization and summarization of probe values. Standard quality controls based on Affymetrix original methods including average background, scale factor, number of genes called present, 3´ to 5´ ratios computed from the MAS 5.0 algorithm and probe-level models (PLM) based on fitting a model for probe values and analyzing its residuals (Relative Log Expression and Normal Unscaled Standard Error) were performed. Arrays that did not show the minimum acceptable quality based on these standard quality controls were discarded. After quality control, the mean expression of each probe set in controls was compared with that of the infected samples to summarize the comparison between controls and the infected samples. Microarray data analysis was done using the free statistical language R and the libraries developed by the Bioconductor Project (http://www.bioconductor.org). In order to deal with the multiple testing issues derived from the fact that many tests (one per gene) were performed simultaneously, p-values were adjusted to obtain strong control over the false discovery rate using the Benjamini and Hochberg method [31]. All the microarray data were deposited at the NCBI Gene Expression Omnibus (GEO) under the platform accession number GPL3533 and the series number GSE15766.

### Sequence analysis

Gene ontology (GO) assignments were retrieved from the GeneChip® Porcine Genome Array (Affymetrix) and verified by searching the Entrez (http://www.ncbi.nlm.nih.gov/sites/gquery) and Gene ontology (http://www.geneontology.org/) databases. The gene ontology (GO) enrichment analysis was performed with GOstats package [32]. For each GO category of interest, entries in the array were compared with results of differentially expressed genes by *χ*2-test (p = 0.01).

### Domestic pigs and sample preparation

Six 9-weeks-old pathogen-free male pigs were randomly distributed into two experimental groups with three animals each, infected and uninfected. Pigs were experimentally infected with *A. phagocytophilum* by intravenous inoculation (iv) of ISE6 tick cell cultures infected with the human NY-18 isolate of *A. phagocytophilum*[33,34]. Pigs were each inoculated with one T-25 flask of *A. phagocytophilum*-infected ISE6 tick cells (11-15% infection, as determined by detection of intracellular morulae in stained cytospin cell smears; Hema-3 Stain, Fisher Scientific, Middletown, VA, USA) at days 0 and 36 of the experiment. Control pigs were inoculated with control uninfected tick cells. Uninfected and infected cultures were centrifuged at 1,000 x g for 5 min and resuspended in L-15B medium without fetal bovine serum and antibiotics in a final iv dose of 1 x 10^7^ cells/2 ml. All pigs were monitored for infection by recording clinical signs, PCR of blood samples, examination of stained blood films and by *Anaplasma* serology at days 0 (before first inoculation), 7, 15, 36 (before second inoculation), 47 and 62. At day 62, pigs were euthanized by a licensed veterinarian and subjected to gross necropsy examination. Animals were cared for in accordance with standards specified in the Guide for Care and Use of Laboratory Animals and approved by ethical committee for animal care and experimentation (No. 10/397354.9/11).

### Detection of *A. phagocytophilum* in experimentally infected pigs by PCR

DNA was extracted from pig blood samples using TriReagent (Sigma, St. Louis, MO, USA) following manufacturer’s recommendations. *A. phagocytophilum* infection levels were characterized by *msp4* PCR using the iQ5 thermal cycler (Bio-Rad, Hercules, CA, USA) as described previously using oligonucleotide primers MAP4AP5: 5’-ATGAATTACAGAGAATTGCTTGTAGG-3’ and MSP4AP3: 5’-TTAATTGAAAGCAAATCTTGCTCCTATG-3’) in a 50-μl volume PCR (1.5mMMgSO4, 0.2mM dNTP, 5XGoTaq reaction buffer, 5u GoTaqDNA polymerase) (Promega, Madison, WI, USA) [2]. Negative control reactions were performed with the same procedures, but adding water instead of DNA to monitor contamination of the PCR. PCR products were electrophoresed on 1% agarose gels to check the size of amplified fragments by comparison to a DNA molecular weight marker (1 kb DNA Ladder, Promega). Amplified fragments were resin purified (Invitrogen, Carlsbad, CA, USA) for sequencing both strands by double-stranded dye-termination cycle sequencing (Secugen SL, Madrid, Spain). The *msp4* coding region was used for sequence alignment. Multiple sequence alignment was performed using the program DNA Baser (Heracle BioSoft S.R.L., Pitesti, Romania).

### Detection of anti-*A. phagocytophilum* antibodies in experimentally infected pigs by ELISA

Serum samples were tested for IgG antibodies by means of an in-house indirect ELISA using the *A. phagocytophilum* (NY-18) recombinant MSP4 protein as antigen and protein G horseradish peroxidase as a conjugate using the protocol described by Araújo et al. [35] with some modifications. Briefly, 96-well plates (MaxiBinding, SPL Life sciences, Korea) were coated overnight at 4°C with 0.4 μg/ml of MSP4, diluted in carbonate-bicarbonate phosphate buffer. Plates were blocked for 1 hr at 37°C with 140 μl/well of a solution containing 5% skim milk with phosphate buffered saline and 0.05% Tween-20 (PBST). Sera were added directly on plate (100 μl/well) at a dilution of 1:100 in PBST and incubated for 1 hr at 37°C. Plates were then washed five times with PBST, and Protein G (Sigma Aldrich, Saint Louis, USA) was added (100 μl/well) at a dilution of 1:1,000 in PBST and incubated at 37°C for 1 hr. After five washes with PBST, the chromogen/substrate o-phenylene diamine dihydrochloride (OPD; Sigma)/H_2_O_2_ was added. The reaction was stopped with 50 μl/well of sulphuric acid (H_2_SO_4_; 3N), and the optical density (OD) was measured in a spectrophotometer at 450 nm. White-tailed deer and cattle sera positive to *Anaplasma* were included as controls. Antibody titers in experimentally infected and control pigs were expressed as the OD_450nm_ (OD_pig sera_ - OD_PBS control_) and compared between inoculated and control groups by ANOVA test (P = 0.05).

### Detection of TNF-alpha, IL-1 beta and IL-8 in experimentally infected pigs by ELISA

The serum levels of TNF-alpha, IL-1 beta and IL-8 (ELISA kits; RayBiotech Inc., Norcross, GA, USA) were determined at days 0, 7, 15, 33, 36, 47 and 62 in infected and control pigs following manufacturer’s recommendations. Briefly, 100 μl of each standard recombinant porcine cytokine (RayBiotech) and pig serum samples were added to 96-well plates (MaxiBinding, SPL Life sciences, Korea) and incubated for 2.5 hrs. Plates were washed 4 times with 1x wash solution, inverted and cleaned with paper towels. Then, 100 μl of biotinylated anti-pig antibody (RayBiotech) were added to each well, incubated for 1 hr and washed as described before. Hundred μl of horseradish peroxidase labeled streptavidin solution (RayBiotech) were added to each well, incubated for 45 min and washed as before. Finally, 100 μl of 3,3’,5,5’-tetramethylbenzidine (TMB) One-Step substrate reagent (RayBiotech) were added to each well, incubated for 30 min in the dark and 50 μl of stop solution (0.2 M sulfuric acid) were added to each well before reading the plate at OD_450nm_ immediately. All incubations were done at room temperature with gentle shaking. The mean OD_450nm_ was calculated for each set of duplicate standards, controls and samples and the average zero standard OD_450nm_ was subtracted. The standard curve was plotted and used to calculate serum cytokine concentrations in infected and control pigs. Infected to uninfected ratios were compared between infected and control pigs by Student’s *t*-test (P = 0.05).

### Buffy coat cell composition in experimentally infected pigs

Buffy coat was obtained by centrifugation of 10 ml of heparin-treated blood at 200xg for 10 min. The Buffy coat was removed and resuspended in 5 ml PBS. Cell suspension (100 μl) was then treated with 1 ml of BD-FACS lysing solution (Becton Dickinson, Madrid, Spain), centrifuged and resuspended in 500 μl PBS. The analyses of cell subpopulations were performed using a FACScalibur (Becton Dickinson) flow cytometer. Subpopulation were gated and counted by their characteristic forward and side scatter. Results were compared between inoculated and control pigs by ANOVA test (P = 0.05).

### Analysis of mRNA levels by real-time RT-PCR analysis

Real-time RT-PCR was performed on RNA samples from naturally and experimentally infected and uninfected pigs with gene specific primers (Table 1) using the iScript One-Step RT-PCR Kit with SYBR Green and the iQ5 thermal cycler (Bio-Rad, Hercules, CA, USA) following manufacturer's recommendations. A dissociation curve was run at the end of the reaction to ensure that only one amplicon was formed and that the amplicon denatured consistently in the same temperature range for every sample [36]. The mRNA levels were normalized against porcine cyclophlilyn, beta-actin and glyceraldehyde 3-phosphate dehydrogenase (GAPDH) using the genNorm method (ddCT method as implemented by Bio-Rad iQ5 Standard Edition, Version 2.0) [37]. In all cases, the mean of the duplicate values was used and data from infected and uninfected animals were compared using the Student’s *t*-test (P = 0.05) or ANOVA test (P = 0.05) for wild and experimental pigs, respectively.

**Table 1:** Primer sets and real-time PCR conditions used for analysis of differentially expressed genes

**Gene description**	**Genbank accession number**	**Upstream/downstream primer sequences (5´-3´)**	**PCR conditions**^**a**^
Interleukin 1 receptor accessory protein-like 1 (*IL1RAPL1*)	NG_008292 CN163387	IL1-L: GTTGTCATTTCGCCAAACCT IL1-R: GCCTATGACCGATGGCTTTA	58°C, 30 sec/72 °C, 30 sec
T-cell receptor alpha chain (*TCR-alpha*)	AB087958.1	TcellR-L: TTCTGACCCTGGGGACTATG TcellR-R: GAGAAGCCATGCTGTTGGT	58°C, 30 sec/72°C, 30 sec
Gap junction protein alpha 1 (*GJA1*)	BC105464.1 CK465005	GAP-L: TGCAATGAAGCTGAACATGA GAP-R: TGGAATGCAAGAGAGGTTGA	58°C, 30 sec/72°C, 30 sec
Thrombospondin 4 (*TSP-4*)	XM_001926236 BM190304	TROMB4-L: GGGCAAGGTTTTTGTTCTGA TROMB4-R: TCATAGGGGTCCAGCACTTC	60°C, 30 sec/72°C, 30 sec
Beta-actin	DQ845171	SusBetActin-L: GACATCCGCAAGGACCTCTA SusBetActin-R: ACACGGAGTACTTGCGCTCT	60°C, 30 sec/72°C, 30 sec
Glyceraldehyde 3-phosphate dehydrogenase (*GAPDH*)	AF069649	GADPHSus-L: CCAGAACATCATCCCTGCTT GADPHSus-R: GTCCTCAGTGTAGCCCAGGA	60°C, 30 sec/72 °C, 30 sec
Cyclophilin	AY008846	SSCYCLOPHILIN-L: AGCACTGGGGAGAAAGGATT SSCYCLOPHILIN-R: CTTGGCAGTGCAAATGAAAA	55 °C, 30 sec/72 °C, 30 sec

^a^PCR conditions are shown as annealing/extension in real-time RT-PCR analysis.

## Results

### Gene expression in pigs naturally infected with *A. phagocytophilum*

All infected wild pigs contained a single *A. phagocytophilum* 16S rDNA and *groESL* genotype. The 16S rDNA sequence was identical to the sequence of the USG3 strain [GenBank: AY055469] originally isolated from a dog infected by feeding infected *I. scapularis* ticks, as well as to strains obtained from patients diagnosed with human granulocytic anaplasmosis (HGA) [38]. The sequence of the *groESL* locus was identical to that identified previously in wild boar, human and *I. ricinus* samples in Slovenia [GenBank: AF033101 and EU246961] [15].

Of the 20,201 *S. scrofa* genes that were analyzed in the microarray, 942 showed significant (P < 0.05) differences between infected and control samples (936 upregulated and 6 down regulated) and 61 of them had >2 fold changes in expression in wild pigs (Table 2). Of these genes, 56 were upregulated and 5 were down regulated in infected animals (Table 2).

**Table 2:** Gene ontology and description of significant differentially expressed genes (P < 0.05; > 2 fold change)

**Affymetrix ID**^**1**^	**Genbank accession number**	**Fold Change**^**2**^	**SD**^**3**^	**P-value**^**4**^	**Description**^**5**^	**GO Molecular funtion**^**6**^	**GO Biological process**^**7**^
Ssc.30381.1.A1_at	CO991016	361.988	84.148	0.039	Unknown		
Ssc.17891.1.A1_at	CF175823	29.073	8.518	0.029	Unknown		
Ssc.13408.1.A1_at	BI405159	19.229	5.72	0.030	Unknown		
Ssc.10537.1.A1_at	BF711416	15.897	1.497	0.002	Unknown		
Ssc.29577.1.A1_at	CO940471	14.279	0.259	0.006	Unknown		
Ssc.24631.1.S1_at	CK461650	11.635	1.792	0.007	Formin 1	Protein binding	Cell adhesion
Ssc.31062.1.S1_at	AJ663560	8.047	1.593	0.021	Unknown		
Ssc.28701.1.S1_at	BG895814	8.008	0.889	0.015	Sorbin and SH3 domain isoform 2, transcript variant 14	Receptor activity	Signaling pathway
Ssc.29538.1.A1_at	CO941727	7.984	0.567	0.020	Unknown		
Ssc.10128.1.A1_at	BI399899	7.217	1.986	0.039	similar to *H. sapiens* SIX homebox 4	Unknown	Unknown
Ssc.16289.1.A1_at	U15437.1	5.927	1.945	0.047	Ig heavy chain variable VDJ region	Protein binding	Immune response
Ssc.16269.1.S1_at	U15523.1	2.919	0.594	0.047	Ig heavy chain variable VDJ region	Protein binding	Immune response
Ssc.15942.5.A1_x_at	U38202.1	2.571	0.398	0.044			
Ssc.17872.1.A1_at	CF175649	5.731	1.428	0.047	COUP transcription factor 1 (*COUP-TF1*)	Transcription factor	Signaling pathway
Ssc.31126.1.A1_at	CO942136	5.391	0.792	0.035	Unknown		
Ssc.29622.1.A1_at	CO942607	4.96	0.634	0.009	Unknown		
Ssc.17942.1.A1_at	CF176409	4.605	0.287	0.020	Unknown		
Ssc.31069.1.A1_s_at	BF712013	4.578	0.478	0.014	DAZ interacting protein 3, zinc finger	Protein binding	Ubiquitin-dependent protein catabolic process
Ssc.6157.1.A1_at	BQ597772	3.782	0.555	0.021	Zinc finger protein 521	Unknown	Unknown
Ssc.1411.1.S1_at	BM190304	3.586	0.152	0.013	Thrombospondin 4 (*TSP-4*)	Cation binding, protein binding	Cell adhesion
Ssc.7524.1.A1_at	BQ599075	3.397	0.719	0.033	Sk/Dkk-1 protein precursor	Protein binding	Signaling pathway
Ssc.8931.1.A1_at	BI398736	3.336	0.818	0.037	Angiopoietin-like protein 2 (*Angptl2*)	Unknown	Signaling pathway
Ssc.13693.1.A1_at	BQ603203	3.313	0.589	0.026	Unknown		
Ssc.4707.1.A1_at	BI118246	3.271	0.917	0.049	*H. sapiens* kit ligand (*KITLG*)	Protein binding	Cell adhesion
Ssc.13265.1.A1_at	BQ605073	3.236	0.602	0.029	Unknown		
Ssc.7967.1.A1_at	BQ599891	3.153	0.663	0.033	Unknown		
Ssc.8871.2.A1_at	CK457442	2.929	0.711	0.043	Cyclin-dependent kinase inhibitor 1C (*CDKN1C*)	Protein binding	Signaling pathway
Ssc.20473.2.S1_at	CK456061	2.909	0.139	0.001	Unknown		
Ssc.20452.1.S1_at	BX670488	2.890	0.616	0.035	Keratin associated protein 26-1	Protein binding	Cell structure
Ssc.29030.1.S1_at	CO988330	2.838	0.499	0.032	Unknown		
Ssc.26632.1.S1_at	CN155689	2.813	0.333	0.030	Tripartite motif protein 32	Protein binding	Cell differentiation, ubiquitin-dependent protein catabolic process
Ssc.24221.2.A1_at	BI181166	2.805	0.302	0.007	NADH-ubiquinone oxidoreductase	Enzymatic activity	Cell metabolism
					18 kDa subunit		
Ssc.428.10.S1_at	AB087975.1	2.767	0.181	0.027	T cell receptor alpha chain (*TCR-alpha*)	Receptor activity	Immune response
Ssc.17790.1.S1_at	AB087958.1	2.334	0.369	0.031	T cell receptor alpha chain (*TCR-alpha*)	Receptor activity	Immune response
Ssc.18884.1.A1_at	CF365209	2.67	0.083	0.008	Unknown		
Ssc.25538.1.S1_at	BX918287	2.597	0.377	0.033	Zinc finger protein 502	Transcription factor	Transcription
Ssc.26587.1.A1_at	CN154795	2.592	0.334	0.030	Unknown		
Ssc.20172.1.A1_at	BX676733	2.547	0.347	0.027	Tumor endothelial marker 8 isoform 3	Protein binding, receptor activity	Cell adhesion
Ssc.7090.1.A1_at	NM_214233.1	2.465	0.435	0.026	Thioltransferase (*GLRX1*)	Enzymatic activity	Stress
Ssc.13474.1.A1_at	BQ602423	2.454	0.405	0.022	Unknown		
Ssc.8511.1.A1_at	BF703957	2.449	0.275	0.009	*Sus scrofa* mRNA,clone: OVRM10011A06, expressed in ovary	Unknown	Unknown
Ssc.30148.1.A1_at	CO987207	2.341	0.208	0.048	Rho-related BTB domain containing 3 (*RHOBTB3*)	Protein binding, receptor activity	Signaling pathway, ubiquitin-dependent protein catabolic process
Ssc.22336.1.S1_at	CF793417	2.302	0.366	0.023	Homeobox protein Hox-B7 (*Hox-2C*)	Transcription factor, protein binding	Transcription
Ssc.1377.2.S1_at	BI343023	2.264	0.19	0.016	Integrin alpha-8 (*ITGA8*)	Cation binding, protein binding, receptor activity	Cell differentiation, cell adhesion, signalling pathway
Ssc.29167.1.A1_at	CO950916	2.198	0.272	0.030	Rho GTPase activating protein 5	Protein binding	Cell adhesion
Ssc.13363.1.A1_at	BI404946	2.188	0.31	0.038	Ubiquitin carboxyl-terminal hydrolase 24	Protein binding	Ubiquitin-dependent protein catabolic process
Ssc.17370.1.A1_at	BX665583	2.186	0.266	0.014	Adrenergic, alpha-1B-, receptor (*ADRA1B*)	Protein binding, receptor activity	Signaling pathway
Ssc.22210.2.S1_at	CF788693	2.176	0.298	0.040	Unknown		
Ssc.29565.1.A1_at	CO942018	2.168	0.308	0.025	Unknown		
Ssc.19407.1.A1_at	CF359796	2.157	0.016	0.015	Unknown		
Ssc.28265.1.A1_at	CN025977	2.143	0.376	0.035	Unknown		
Ssc.26179.1.S1_at	BX922022	2.123	0.101	0.005	Midnolin (*MIDN*)	Protein binding	Transcription
Ssc.4848.1.S1_at	CF789770	2.123	0.182	0.031	Calponin 3, acidic, transcript variant 1	Cation binding, protein binding	Unknown
Ssc.20453.1.S1_at	BX675824	2.092	0.228	0.012	Laminin receptor 1	Receptor activity	Unknown
Ssc.14354.1.A1_at	BQ601965	2.079	0.093	0.047	HHEX gene for hematopoietically expressed homeobox	Transcription factor	Cell differentiation, signaling pathway
Ssc.942.1.S1_at	CK465005	2.061	0.165	0.012	Gap junction protein, alpha 1 (*GJA1*)	Protein binding	Cell adhesion, signaling pathway, immune response
Ssc.26933.1.S1_at	CN163387	2.036	0.000	0.007	Interleukin 1 receptor accessory protein-like 1 (*IL1RAPL1*)	Receptor activity	Immune response
Ssc.30263.1.A1_at	CO989398	2.033	0.300	0.039	Unknown		
Ssc.16566.1.S1_at	BF078197	2.025	0.271	0.025	Lactase phlorizinhydrolase	Cation binding	Cell metabolism
Ssc.9748.1.A1_at	BI398784	−3.914	1.462	0.033	Unknown		
Ssc.30189.1.A1_at	CO987781	−4.078	1.800	0.038	Pig DNA sequence from clone CH242-94D11 on chromosome 7	Unknown	Unknown
Ssc.8698.1.S1_at	CN163671	−10.246	0.151	0.010	Cadherin 11, type 2, OB-cadherin (osteoblast)	Cation binding, protein binding	Cell adhesion
Ssc.18866.1.A1_at	CF365015	−10.551	1.242	0.003	Unknown		
Ssc.29259.1.A1_at	CO953119	−14.935	2.417	0.048	Zinc finger protein 567	Transcription factor	Transcription

^1^ Affymetrix ID identifies the probe based on Affymetrix accession number.

^2^ Fold change is the normalized average log2 ratio of infected/uninfected wild pigs (negative values denote dowregulation in infected animals).

^3^ SD is the standard deviation of the normalized average log2 ratio.

^4^ P-value determined from the average log2 ratio.

^5^ Description indicates a short description of the gene.

^6^ GO molecular function is the function of the gene according to GO entries.

^7^ GO biological process indicates the gene process according to GO entries.

Gene ontology (GO) could be assigned to 32 of the differentially expressed genes (Table 2). The differentially expressed genes in wild pigs infected with *A. phagocytophilum* included those with cation binding, protein binding, transcription factor, enzymatic activity and receptor activity protein function involved in cell differentiation, adhesion, metabolism and structure, signaling pathway, transcription, stress, immune response and catabolic processes (Table 2). The most frequently represented protein function and biological process GO assignments were significantly overrepresented among genes differentially expressed in wild pigs and contained genes upregulated in response to *A. phagocytophilum* infection (Table 3). Among them, the highest GO enrichment for molecular function and biological process occurred for protein binding and signaling pathway genes, respectively (Table 3).

**Table 3:** **Gene ontology enrichment analysis of genes differentially expressed in wild pigs naturally infected with* A. phagocytophilum ***

**GO category**	**Represented on the microarray (%)**^**a**^	**Represented among differentially expressed genes (%)**^**b**^
**Molecular function**		
Cation binding	67 (2.4)	5 (15.6)*
Protein binding	14 (0.5)	20 (62.5)*
Transcription factor	10 (0.4)	5 (15.6)*
Receptor activity	10 (0.4)	8 (25.0)*
**Biological process**		
Catabolic process	108 (3.8)	3 (9.4)*
Immune response	23 (0.8)	4 (12.5)*
Cell adhesion	20 (0.7)	8 (25.0)*
Signaling pathway	13 (0.5)	10 (31.2)*
Cell differentiation	7 (0.2)	3 (9.4)*

^**a**^Of the 20,201 *S. scrofa* genes analyzed in the microarray, 2,840 had GO assignments and were used for GO enrichment analysis.

^**b**^Of the 61 genes that showed significant (P ≤ 0.05) ≥ 2 fold changes in expression in infected wild pigs, 32 had GO assignments and were used for GO enrichment analysis. For genes with multiple GO assignments, each category was included in the analysis. For each GO category of interest, entries in the array were compared with results of differentially expressed genes by *χ*2–test (*α < 0.01).

**Figure 1:**
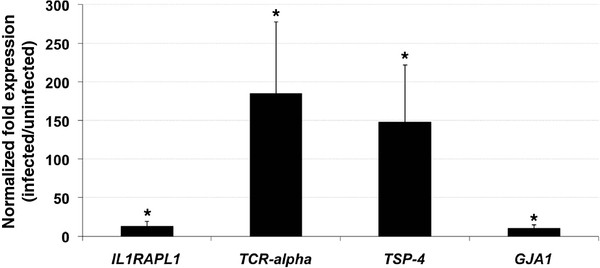
**Relative expression of immune response genes in naturally* A. phagocytophilum *-infected and uninfected wild pigs.** The expression of selected genes was quantified by real-time RT-PCR in samples of infected (N = 3) and uninfected control pigs (N = 3). Amplification efficiencies were normalized against porcine cyclophlilyn, beta-actin and * GAPDH * and infected to uninfected average ± S.D. mRNA ratios determined. In all cases, the mean of duplicate values was used and data from infected and uninfected animals were compared using the Student`s *t*-test (*P < 0.05).

The immune response was among the biological processes significantly overrepresented in genes upregulated in response to *A. phagocytophilum* infection (Table 3). Thus, the immune response genes upregulated in response to *A. phagocytophilum* infection, interleukin 1 receptor accessory protein-like 1 (*IL1RAPL1*), T-cell receptor alpha chain (*TCR-alpha*), thrombospondin 4 (*TSP-4*) and Gap junction protein alpha 1 (*GJA1*), were selected for confirmation of microarray hybridization results by real-time RT-PCR. The real-time RT-PCR analysis confirmed the results of the microarray hybridization and demonstrated that the immune response genes *IL1RAPL1*, *TCR-alpha*, *TSP-4* and *GJA1* were upregulated in infected animals (Figure 1).

### Gene expression in pigs experimentally infected with *A. phagocytophilum*

**Figure 2:**
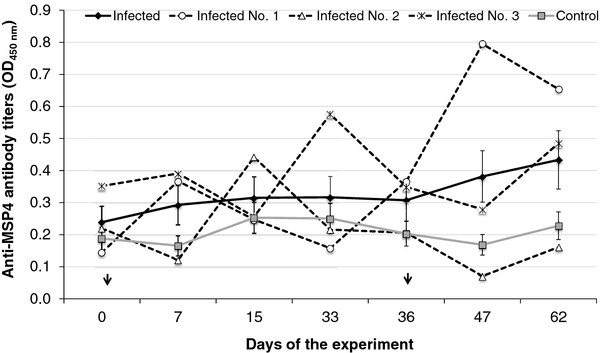
**Detection of anti-* A. phagocytophilum *MSP4 antibodies in experimentally infected and control pigs.** Antibody titers were determined by ELISA, expressed as the average ± S.E. OD_450nm_ (OD_pig sera_ - OD_PBS control_) and compared between infected and control pigs by ANOVA test (P > 0.05). OD_450nm_ values for each infected pig are also shown. Arrows show time of pig inoculation with infected and uninfected tick cells.

In experimentally infected pigs, *A. phagocytophilum* DNA was detected by *msp4* PCR in blood samples collected at 15 (in all 3 pigs), 36 (before second inoculation in pigs No. 1 and No. 2), and 62 (in pig No. 1 only) days post-infection (dpi) in pigs inoculated with infected cells but not in control pigs. The *A. phagocytophilum msp4* amplicons from pig blood were sequenced and corresponded to the NY-18 isolate sequence (Genbank accession number JQ522935). Infected and uninfected pigs did not show clinical signs or *A. phagocytophilum* morulae in stained blood films. Significant differences were not observed in anti-*A. phagocytophilum* MSP4 antibodies between pigs inoculated with infected cells and controls (P > 0.05; Figure 2). However, peaks in anti-MSP4 antibody titers were detected at 33 and 47 dpi in pigs No. 3 and No. 1, respectively (Figure 2).

**Figure 3:**
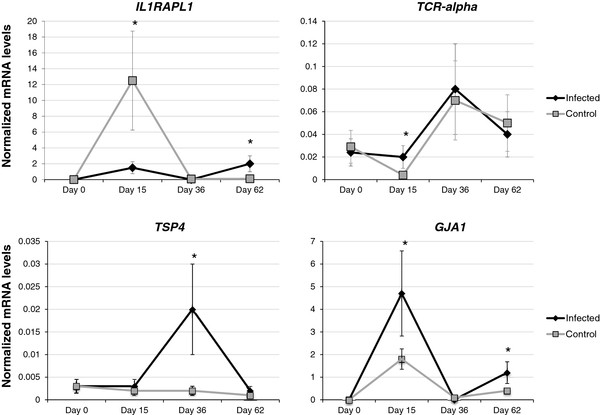
**Expression of immune response genes in experimentally* A. phagocytophilum *-infected and uninfected domestic pigs.** The expression of selected genes was quantified by real-time RT-PCR in samples of infected (N = 3) and uninfected control pigs (N = 3). Amplification efficiencies were normalized against porcine cyclophlilyn, beta-actin and * GAPDH * and shown in arbitrary units as average ± S.D. mRNA levels. In all cases, the mean of duplicate values was used and data from infected and uninfected animals were compared using the ANOVA *t*-test (*P < 0.05).

Buffy coat cell composition did not change during the experiment and was similar between infected (lymphocytes, 37.80 ± 0.13%; monocytes, 10.51 ± 0.02%; granulocytes, 49.56 ± 0.16%) and uninfected (lymphocytes, 36.16 ± 0.11; monocytes, 10.21 ± 0.01; granulocytes, 49.43 ± 0.13%) pigs (P > 0.4). The immune response genes upregulated in response to *A. phagocytophilum* infection in naturally infected pigs (*IL1RAPL1*, *TCR-alpha*, *TSP-4* and *GJA1*) were selected to characterize the mRNA levels at different dpi by real-time RT-PCR in experimentally infected pigs (Figure 3). The results showed that *TCR-alpha* and *GJA1* were upregulated in infected pigs at 15 dpi when compared to control animals. *TSP-4* was upregulated at 36 dpi only while *IL1RAPL1* and *GJA1* were upregulated at 62 dpi (Figure 3). The highest mRNA levels for immune response genes at the end of the experimental infection (62 dpi) were found in the only infected pig in which pathogen DNA was detected by PCR (pig No. 1).

### Serum IL-1 beta, IL-8 and TNF-alpha levels in pigs experimentally infected with *A. phagocytophilum*

**Figure 4:**
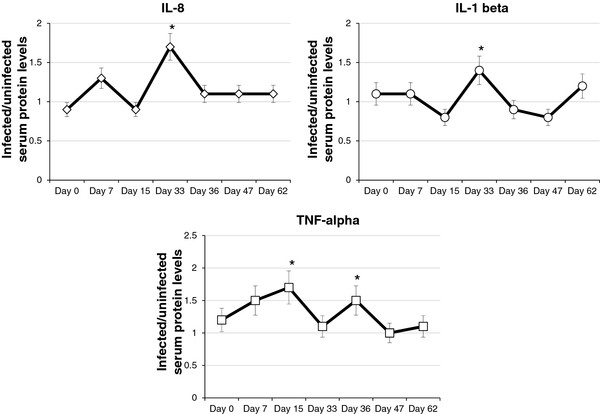
**Serum IL-8, IL-1 beta and TNF-alpha levels in experimentally infected pigs.** Cytokine levels were determined by ELISA in the sera from infected and uninfected control pigs and infected to uninfected average ± S.D. ratios determined. Results were compared between infected and control pigs by Student’s *t*-test (*P ≤ 0.05).

Serum IL-1 beta, IL-8 and TNF-alpha levels were transiently higher in infected pigs when compared to uninfected controls (Figure 4). Significant (P < 0.05) infected to uninfected ratio for serum protein levels were obtained for IL-1 beta and IL-8 at 33 dpi and for TNF-alpha at 15 and 36 dpi (Figure 4). These protein levels were equivalent in infected animals to 3.73 ± 0.00 pg/ml (IL-1 beta), 2.18 ± 0.00 pg/ml (IL-8), 370.13 ± 0.00 pg/ml (TNF-alpha at 15 dpi) and 2.01 ± 0.00 pg/ml (TNF-alpha at 36 dpi). In uninfected control animals, protein levels at the same time points were bellow ELISA detection limits.

## Discussion

Molecular evidence suggested that wild pigs could be involved in the natural cycle of *A. phagocytophilum* in some regions [14-16,21]. The results of sequence analyses suggested that the *A. phagocytophilum* strain identified in wild pigs might be similar to those causing disease in dogs and humans, thus reinforcing the possible role of pigs in the epidemiology of HGA in these regions [15,38,39].

**Figure 5:**
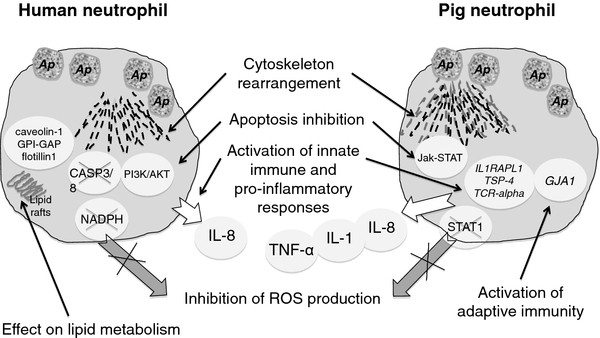
**Effect of* A. phagocytophilum *infection on host cells.*** A. phagocytophilum * (*Ap*) infection causes cytoskeleton rearrangement required for infection, but in pigs it may also promote phagocytosis and autophagy for effective pathogen clearance. * Ap * delays the apoptotic death of neutrophils to increase infection, but different and complementary mechanisms may operate in human and pig cells. Pathogen infection stimulates innate immune and pro-inflammatory responses in both humans and pigs. IL-8 is likely secreted by infected neutrophils but monocytes, rather than neutrophils, are probably responsible for proinflammatory IL-1 beta and TNF-alpha cytokine production. The expression of genes involved in adaptive immunity was not impaired in pigs. ROS production is inhibited by pathogen infection of human neutrophils but although this mechanism was not found in pigs, upregulation of TGF-beta in infected pigs may inhibits NO production by suppressing STAT1 activation and accelerating iNOS protein degradation. The effect on lipid metabolism required for pathogen infection of human neutrophils was not found in pigs. Data for human neutrophils was obtained from the recent review by Severo et al. [43]

The overall effect of *A. phagocytophilum* on pig gene expression was low as only 4.7% (942/20,201) of the genes analyzed in the microarray were differentially expressed in pathogen-infected animals (P < 0.05) and only 61 genes (0.3%; 61/20,201) showed >2 fold difference between infected and control animals. Interestingly, 9 of the 61 (15%) differentially expressed genes in naturally infected pigs were related to cytoskeleton structure and function. Phagocytosis and autophagy are among the first lines of defense against bacterial infections and require a dramatic rearrangement of the cytoskeleton for internalization of invading microbes [40]. The expression of genes such as *GJA1*, integrin alpha-8, *TSP-4*, formin 1, Rho GTPase activating protein 5, keratin associated protein 26–1, calponin 3 and laminin receptor 1 was upregulated, while the expression of cadherin 11 was down regulated in *A. phagocytophilum*-infected wild pigs, thus suggesting an effect of pathogen infection on cytoskeleton rearrangement. It has been suggested that *A. phagocytophilum* affects actin reorganization to facilitate cell invasion but reduces neutrophil phagocytosis and subverts autophagy to establish intracellular infection and proliferation [41-43]. Furthermore, a recent study showed that Toll-like receptor signaling usurps components that are traditionally associated with autophagy to increase the efficiency of phagocytosis, thereby providing a link between these two microbial defense mechanisms [44]. Taken together, these results suggested that *A. phagocytophilum* infection of pigs impacted cytoskeleton rearrangement to promote phagocytosis and autophagy, thus resulting in effective pathogen clearance (Figure 5).

*A. phagocytophilum* infection has been shown to delay the apoptotic death of neutrophils [24,43,45,46]. The analysis of gene expression profile in naturally infected pigs did not show an effect on caspases 3 and 8 (CASP3/8) and the PI3K/AKT pathway, which have been linked to *A. phagocytophilum*-induced apoptosis inhibition in human neutrophils [43]. However, the activation of the Jak-STAT pathway that has been shown to occur in *A. phagocytophilum*-infected sheep and pigs may constitutes a new mechanism leading to delay in the apoptotic death of neutrophils in these species [47] (Figure 5). Reactive oxygen species (ROS) production is inhibited by *A. phagocytophilum* through modulation of NADPH oxidase assembly and/or regulation of gene expression in human cells [43], a mechanism that was not found in pigs. However, upregulation of TGF-beta in infected pigs [47] may inhibits NO production in neutrophils by suppressing STAT1 activation and accelerating iNOS protein degradation [47,48]. The effect of *A. phagocytophilum* on lipid metabolism required for pathogen infection of human neutrophils [25,43] was also not found in pigs. However, some of these discrepancies may be explained by the fact that results in pigs were obtained using RNA from Buffy coats and not purified neutrophils or cell cultures, which may produce a masking effect of other leukocyte mRNAs.

Our group is interested in the characterization of the host immune response to intracellular bacteria [29,49-52]. The infection with *A. phagocytophilum* has been shown to stimulate innate immune and pro-inflammatory responses [43,45,46,53]. However, experiments in mice have shown that *A. phagocytophilum* infection may be controlled, even in the absence of innate immune effectors [54,55]. In sheep and horses, evidence suggests that *A. phagocytophilum* infection triggers innate immune responses while impairing adaptive immunity [29,56], a factor that could contribute to pathogenicity in these species.

Analysis of gene expression in naturally and experimentally infected pigs suggested that *A. phagocytophilum* infection increased innate immunity by up regulation of *IL1RAPL1TSP-4* and *TCR-alpha* (Figure 5). Furthermore, kinetics of mRNA levels in experimentally infected pigs showed an early, transient up regulation of immune response genes, probably coinciding with the first bacteremia of the acute infection phase [57]. Up regulation of *IL1RAPL1* and *TSP-4* may increase the innate immune proinflammatory response through improved signal transduction and secretion of IL-1 and IL-8, respectively [58,59]. T lymphocytes use their TCR as a pattern recognition receptor to sense the presence of infection and produce after activation proinflammatory cytokines such as TNF-alpha [60]. In experimentally inoculated pigs, IL-1 beta, IL-8 and TNF-alpha serum levels were transiently higher in infected animals when compared to controls, thus corroborating the stimulation of proinflammatory responses suggested by gene expression studies in *A. phagocytophilum*-infected pigs (Figure 5). IL-8 secretion in response to *A. phagocytophilum* infection in human cells leads to neutrophils recruitment [43]. Although IL-1 and TNF-alpha levels have not been found to be elevated in HGA patients, higher mRNA or serum levels have been observed in horses and sheep, for which *A. phagocytophilum* is also pathogenic [61]. *In vitroA. phagocytophilum* infection of human peripheral blood lymphocytes and monocytes induce transient mRNA expressions and protein secretion of IL-1 beta and TNF-alpha [61]. These studies suggested that although IL-8 is likely secreted by neutrophils, monocytes, rather than neutrophils, are responsible for proinflammatory IL-1 beta and TNF-alpha cytokine production [61,62]. The expression of genes involved in adaptive immunity was not impaired. In fact, the expression of *GJA1*, a member of the connexin gene family with a role in innate and adaptive immunity through the regulation of phagocytosis by macrophages and the host response to bacterial infection [63], was upregulated in infected pigs. The activation of the Jak-STAT pathway associated with *A. phagocytophilum* infection in sheep and pigs may results in immune development to aid in pathogen control [47].

The experimental infection with *A. phagocytophilum* demonstrated that pigs are susceptible to pathogen infection. The detection of bacterial DNA by PCR showed a prepatent period (calculated as the number of days from the time of pig inoculation with infected tick cells to the first day that blood samples were found to be *A. phagocytophilum* positive by PCR) of 15 days, similar to that found in sheep [64] and white-tailed deer [65] but lower than in mice [66] inoculated with *A. phagocytophilum* (NY-18) infected cells. At 36 dpi only two animals were PCR positive and by 47 dpi all animals were negative, suggesting duration of approximately 30 days for the primary bacteremia. However, although only one pig (No. 1) was PCR positive at 62 dpi after the second inoculation, recurrent bacteremias are possible [57]. The weak antibody response detected in infected animals supports a rapid control of pathogen infection. However, similar results were obtained in sheep experimentally inoculated with *A. phagocytophilum* infected cells [64]. The pigs used in this study for microarray analysis were naturally infected with *A. phagocytophilum*. Therefore, it was not possible to establish when animals were infected. Transient up regulation of immune response genes in experimentally infected pigs suggested that naturally infected pigs were also at early infection stages. However, we cannot exclude the possibility that, if pigs become persistently infected even at low infection levels, some of the gene expression profiles described in this study in naturally infected pigs may represent the response of persistently infected animals and may differ from the response during early infection. Persistent *A. phagocytophilum* infection has been documented in sheep [57] and horses [67] and previous studies have shown differences in gene expression profiles between acutely and chronically *A. phagocytophilum*-infected sheep [29].

## Conclusions

These results suggested that pigs are susceptible to *A. phagocytophilum* but control infection, particularly through activation of innate immune responses and cytoskeleton rearrangement to promote phagocytosis and autophagy (Figure 5). Control of *A. phagocytophilum* infection in pigs may results in infection below PCR detection levels or infection clearance, thus contributing to the low percentage of infection prevalence detected for this species in most regions, with a low or no impact as a reservoir host for this pathogen [14,15,20]. The results reported here confirmed in pigs the activation of innate and adaptive immune pathways during *A. phagocytophilum* infection reported in humans and other species (Figure 5). However, this pathogen may uses other mechanisms to circumvent host-cell defenses and establish infection by dowregulating other adaptive immune response genes such as IL-2 and IL-4 and delaying the apoptotic death of neutrophils through activation of the Jak-STAT pathway [47]. These results further expand the existing information on the response of mammalian hosts to *A. phagocytophilum* infection and suggested a role for newly reported genes in the protection of pigs against *A. phagocytophilum*.

## Competing interests

The authors declare that they have no competing interests.

## Authors’ contributions

RCG performed microarray analysis and lab tests, NA, KSS, BB-B, MM and NG collected data and samples, NA, MB and JMP performed lab tests. RCG, NA, MB, JMP, CG and JF analyzed data and performed statistical analysis. JF, KMK and TA-Z conceived the study, JF designed the study, CG supervised part of study, RCG, KMK, TA-Z, CG and JF wrote the manuscript. All authors read and approved the final manuscript.
